# A Hybrid Digital-Signature and Zero-Watermarking Approach for Authentication and Protection of Sensitive Electronic Documents

**DOI:** 10.1155/2014/514652

**Published:** 2014-08-28

**Authors:** Omar Tayan, Muhammad N. Kabir, Yasser M. Alginahi

**Affiliations:** ^1^IT Research Center for the Holy Quran and Its Sciences (NOOR), Taibah University, Madinah 41411, Saudi Arabia; ^2^College of Computer Science and Engineering (CCSE), Department of Computer Engineering, Taibah University, Madinah 41411, Saudi Arabia; ^3^Department of Multimedia and Graphics, Faculty of Computer Systems & Software Engineering, Universiti Malaysia Pahang, Malaysia; ^4^Academic Services, Department of Computer Science, Taibah University, Madinah, Saudi Arabia

## Abstract

This paper addresses the problems and threats associated with verification of integrity, proof of authenticity, tamper detection, and copyright protection for digital-text content. Such issues were largely addressed in the literature for images, audio, and video, with only a few papers addressing the challenge of sensitive plain-text media under known constraints. Specifically, with text as the predominant online communication medium, it becomes crucial that techniques are deployed to protect such information. A number of digital-signature, hashing, and watermarking schemes have been proposed that essentially bind source data or embed invisible data in a cover media to achieve its goal. While many such complex schemes with resource redundancies are sufficient in offline and less-sensitive texts, this paper proposes a hybrid approach based on zero-watermarking and digital-signature-like manipulations for sensitive text documents in order to achieve content originality and integrity verification without physically modifying the cover text in anyway. The proposed algorithm was implemented and shown to be robust against undetected content modifications and is capable of confirming proof of originality whilst detecting and locating deliberate/nondeliberate tampering. Additionally, enhancements in resource utilisation and reduced redundancies were achieved in comparison to traditional encryption-based approaches. Finally, analysis and remarks are made about the current state of the art, and future research issues are discussed under the given constraints.

## 1. Introduction

Recent advancements in information and communication technologies combined with the widespread growth of the Internet have enabled the ease of digital content distribution, communication, and reproduction. Consequently, millions of users from the digital community are able to benefit from the advantages of the fast and simple digital information exchange. However, it is pointed out that such benefits come together in-hand with the problems and threats associated with ensuring digital copyright protection, preventing digital counterfeiting, proof of authenticity, and content-originality verification as an essential requirement largely for online disseminations of sensitive and specialized, formal, legal, financial, and religious content. Essentially, all such digital multimedia contents in the Internet can be classified into images, text, audio, and video, with the challenge being to ensure secure and reliable communications for each media type. This paper is primarily concerned with document integrity and source traceability with regard to widely disseminated digital text resources while reducing resource redundancies from traditional schemes when applied for our target domain. The problem of achieving authenticity and integrity verification for sensitive online text documents/media was presented in the literature as a challenging research problem in [1–15].

Most related studies on cryptography and copyright protection for authentication and integrity protection ignored the performance impact due to the high complexities and relatively large redundant implementation overheads used and particularly when applied for simpler applications that only require confirming authentication and integrity protection (rather than secrecy) of sensitive content [16]. In other cases, such schemes involved the overhead requirement for distributing algorithms and/or keys between communicating parties. For instance, well-known encryption-based digital-signature techniques had offered secrecy during data transmission, applied restrictions on data-access for copyright protection, and were able to detect unauthorized changes to the data. However, most of those schemes had involved large overheads in complex algorithmic computations and in the number of keys required, in addition to the distribution of those keys and algorithms between the communicating parties [16–18].

Other reasons also exit as to why encryption alone cannot provide a completely workable solution in particular applications. For instance, encryption carries overheads in resources and for some applications; it may be preferable to send data with no secrecy or such redundant overhead. Other cases include scenarios when some network management protocols separate confidentiality and integrity functions, rendering encryption alone as inappropriate.

A number of works based on hashing and message digests (MDs) were then proposed for achieving authentication and integrity with reduced overhead as a tradeoff for removing secrecy measures during transmission in order to achieve improved performance when applied in scenarios involving public-key algorithms [16, 19]. However, those works were primarily concerned with* accuracy *(e.g., only confirming authenticity and integrity) of the data rather than with the* performance* overhead incurred. Moreover, hashing approaches had involved the initial problem of exchanging “public-keys” between potentially many communicating parties. It is to the best of our knowledge that only few prior studies had focused on authentication and integrity schemes in the domain of both conflicting requirements (e.g., accuracy and enhanced performance) for those performance-dependent applications involving sensitive electronic documents.

More recently, steganography and watermarking techniques have been found in the literature for embedding hidden marker data in cover media without significantly degrading the quality of the media. Essentially, the hidden watermark serves to identify ownership and to verify its authenticity and integrity or otherwise to detect any modifications to the data. However, watermarking approaches are unable to control access to the data and hence are mainly ideal for applications that require integrity/authenticity verification rather than secrecy in the communications channel. In each of the above approaches (e.g., digital signatures, hashing, and watermarking), the primarily* accuracy*-based requirements were achieved by operating on any media type and sequence of bits (e.g., images, text, and audio bit patterns).

In this work, we focus on confirming authenticity and intact integrity of sensitive text content whose primary motive may compromise the need for secrecy in the communications channel during transmission. The motive here is that it may be required or even desirable that particular sensitive content should be freely propagated via multiple publishers/servers for wider outreach and dissemination. Hence, the well-understood relation between the client(s) and publisher/server now differs from the common one-to-one relation as in e-commerce transactions that had typically involved hashing or encryption algorithms being distributed between two or more known parties. Furthermore, the use of private keys for each (particular) client/receiver (as in public-key cryptosystems) is no longer required or applicable in our system, in which the goal of integrity robustness would require swiftly checking that sample documents from any client browser are authentic and untampered. This paper considers digital-signature and watermarking schemes for our target application domain and proposes a hybrid approach that employs concepts taken from digital-signature and watermarking schemes to achieve our goal. Our approach was evaluated through extensive experiments, with the results demonstrating that our scheme could be optimized for the target application domain of sensitive online texts that require authenticity and integrity verification with no secrecy in the communications channel. Significantly, results from our scheme had demonstrated that our goal could be achieved whilst avoiding the overhead of registering secret keys from all parties with a certification authority (e.g., as in symmetric-key signature schemes) as well as removing the need for separate public and private keys (the need for private keys was completely removed in our approach) for each communicating party (e.g., as in public-key signature schemes).

This paper is organized as follows: Section 2 provides the related work on digital-signatures and watermarking schemes, Section 3 explains the proposed hybrid digital-signature and zero-watermarking based framework, Section 4 discusses the analysis of the proposed framework, and finally Section 5 concludes the paper.

## 2. Related Work

### 2.1. Digital-Signature Schemes

Cryptography is used to protect information during the transmission process in applications that include emails, banking, sales, and corporate/private data. Cryptographic schemes are classified into symmetric-key systems and asymmetric-key systems [20]. Digital signature schemes are based on symmetric-key or asymmetric-key systems and offer effective mechanisms for facilitating content authenticity, integrity, and data-secrecy during transmission. The two most commonly used public-key digital-signature schemes are the Rivest-Shamir-Adleman (RSA) public-key encryption algorithm and the digital-signature algorithm (DSA) [21].

**Figure 1:**
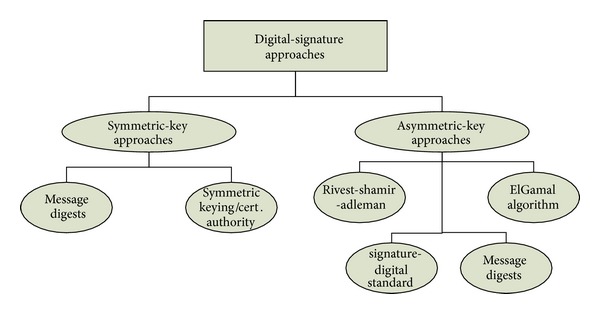
Classification of traditional digital-signature schemes.

The work in [22] presents a theoretical performance analysis of DES and RSA with their working mechanisms. This study presents cases where public-keying schemes are preferred to secret-key systems. In [23], the comparison between different symmetric cryptosystems concluded that DES is the most widely used encryption scheme, with 3DES being the slowest algorithm. In comparison, RC4 required the least memory space for implementation and had minimum simulation times. A summary of some traditional and commercial digital-signature techniques is classified as shown in Figure 1.

A number of works can be found in the literature with contributions mainly associated with limited improvements to the existing digital-signature techniques and algorithms. Examples of improvements developed in the literature include [17, 24–27]. In [24], the ElGamal digital-signature scheme was improved using a random number to increase the difficulty of a third-party obtaining the decipher key. Lui and Li [25] report on computation and communication improvements to a previously enhanced digital-signature scheme in the literature. Reference [27] discusses an efficiency enhancement to the RSA algorithm by speeding up certain array-based computations. Lin and Qiu [17] report on two improved digital-signature schemes based on a previous design of a directed signature scheme. Finally, a number of hybrid approaches had also reported some improvements to the existing and commercial techniques by combining digital signatures with either of watermarking, random numbers, and hash functions [18, 19, 24, 28, 29].

### 2.2. Steganography and Digital-Watermarking Schemes

In the literature, the techniques employed to provide the necessary copyright protection and integrity robustness for digital content are known as digital watermarking. A watermark is a signature or unique logo of an organization or individual who owns the rights to digital content [1] and typically contains information related to the copyrights, ownership, publisher, and document information [2]. Watermarking extends the information in the cover text and becomes an attribute of the watermarked document, in which “the object of communication is the packaging and the hidden message only references that packaging” [3]. Traditionally, digital-watermarking techniques are mainly used to embed identification data into the host cover document, in which the embedded data is a function of the host data/content bit sequences [4, 5, 30, 31]. Security issues of text-watermarking are the characteristic of its specific requirements and features and differ greatly from those of other multimedia watermarking schemes [6]. For example, it is relatively easy to insert watermark data into images as compared with plain text since the images contain plenty of redundant areas allowing the watermark data to be inserted whilst retaining perceptual similarity with the original file [2]. Plain text, on the other hand, has a clear structure and little/no redundant data (as found in the case of many languages including English), which negatively affects both the watermark capacity and security [7], therefore increasing the difficulty involved addressing this research problem.

**Figure 2:**
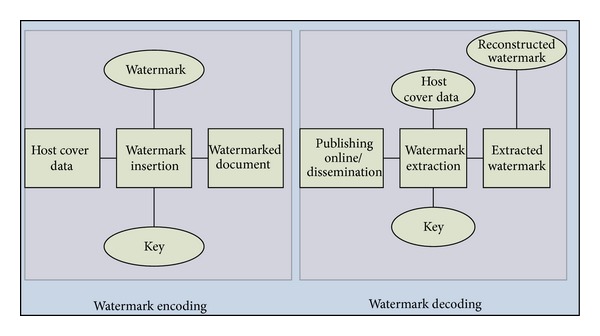
Phases in the watermarking life-cycle.

Some of the objectives of the state of the art in digital text-watermarking can be classified into assuring authenticity and integrity of documents, identifying the origin or publisher/distributer of the contents, usage control, and general protection of documents [3].  Figure 2 outlines the important phases in the life cycle of a generic text-watermarking model.

**Figure 3:**
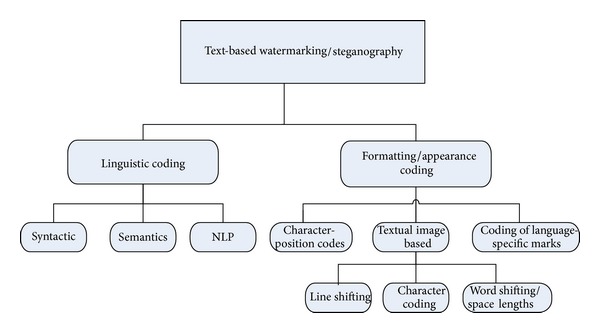
Digital-watermarking classification.

A review of the literature evidences the maturity of watermarking and steganography based techniques in digital natural-language documents and digital text content in some languages including English, Persian, Turkish, and Chinese [4, 8–10], with only fewer techniques presented for the case of other semitic languages such as Arabic electronic texts [7, 8, 11]. Furthermore, watermarking of text documents has been classified into linguistic steganography and nonlinguistic steganography [12]. In the former, the techniques employed would typically manipulate the lexical, syntactic, and semantic properties while trying to preserve the meanings, whilst, in the latter approach, techniques are characterized by the file types and amendments are made to the text by using different text attributes to embed a message. Text-based watermarking has traditionally used shifting techniques or natural-language based watermarking [12]. Three types of text-watermarking shifting codes include line-shift coding, word-shift coding, and feature/character coding, whilst natural-language watermarking involves either of synonym substitutions or semantic transformation techniques which are very language-dependent [12]. On the other hand, the work on [13] classifies text-watermarking techniques into image-based techniques, syntactic-based manipulation, and semantic-based manipulation techniques which involve replacing the original text with alternative words in order to embed a hidden message whilst preserving the meanings as far as possible.  Figure 3 summarizes some of the traditional watermarking techniques found in the literature for the different world languages.

In [6], Zhou et al. classified text-watermarking schemes into four categories of embedding modes: format watermarking, content watermarking, zero watermarking, and binary-image document watermarking [6]. The literature evidences, however, that text-watermarking is a relatively new field as compared with other forms of multimedia with slow development of techniques due to the simplicity and nonredundancy of the text [9]. Comparing fragile, semifragile, and robust watermarking, robust watermarking approaches have attracted attention of more researchers to date [9]. In either case, the designer's choice of watermarking approach should take into consideration the nature/characteristics of the target application since no single optimal scheme exists for all application types [6].

A key requirement for document protection arises with the need for users to confirm authenticity and integrity of the received text [14]. Many traditional text-watermarking techniques based on format-related embedding by modifying text layout and appearances have weak robustness [14]. Such approaches are vulnerable to the detection of the watermark data in the cover text and are more entitled to present themselves more for possible security attacks. Generally, text-watermarks can be attacked in a number of ways, which include inserting, deleting, and rearranging words and phrases [1]. Recently, however, zero-watermarking schemes have been proposed to overcome the problems of weak imperceptibility as well as the tradeoff that exists between robustness and imperceptibility [14, 15]. In such approaches, an attacker's examination of nonoriginal/unnormal formatting codes (causing distortion) in the cover text would be completely removed by eliminating the need for any physical embedding. Here, rather than physically inserting the watermark data, zero-watermarking schemes generate binary patterns during the encoding process by extracting essential characteristics from the host data which are then used in the detection process [14]. It is noted, however, that most of the existing zero-watermarking approaches are designed for image or audio media, with insufficient research conducted using such methods for text documents.

Furthermore, text-watermarking methods found in the literature are very limited and specific to few languages only, in addition to the lack in robustness, integrity, accuracy, and generality [13]. Hence, this work has been motivated by the need to address the deficiencies in text-watermarking, whilst addressing the challenges of generality, integrity, and robustness. In the proposed zero-watermarking approach presented here, no use of steganography is required, since no physical embedding of data is performed on the document. On the contrary, manipulations are performed on the document to determine whether or not the document has been modified and in order to verify the source. The next section describes our proposed hybrid scheme which addresses the above problems by ensuring language independency, invisibility, and robustness and preserves data integrity.

## 3. Proposed Hybrid Digital-Signature and Zero-Watermarking Approach

This paper introduces an implementation of a new design approach for integrity and authentication protection of plain text documents based on zero-watermarking with manipulations also related to digital-signature schemes. The proposed approach resembles digital-signature schemes through the manipulations required at the encoder and decoder as well as through the use of watermark keys/signatures used to verify source authenticity. On the other hand, our approach differs from traditional digital-signature schemes since in our scheme complex encryption operations and their associated overheads are not required during transmission. The goal here is to provide a mechanism for the secure dissemination of critical and sensitive documents in which any physical modification can render the document invalid for the user. Application examples of such requirements are numerous and include formal/official, financial, political, and religious text documents used to prove the original publisher in addition to assuring accuracy and integrity of the data. In the proposed approach, a novel hybrid framework related to digital signatures and zero-watermarking is described.

### 3.1. Description

The proposed algorithm performs a logical embedding of the watermark-data in the cover document. As such, the algorithm does not modify the text in the cover data to embed the watermark, but rather, watermark keys,* W*_KG1_ and* W*_KG2_, are generated based on the* characteristics* of the text document. The Unicode standard is used in the encoder and decoder in order to encode all characters of the main worldwide languages and therefore provide support for worldwide language compatibility. Additionally, the objective of this paper is achieved using a blind watermark-extraction approach, since the original document is not required in the decoding phase and any detected change in the transmitted document/document-under-scrutiny is considered invalid for client use.

**Figure 4:**
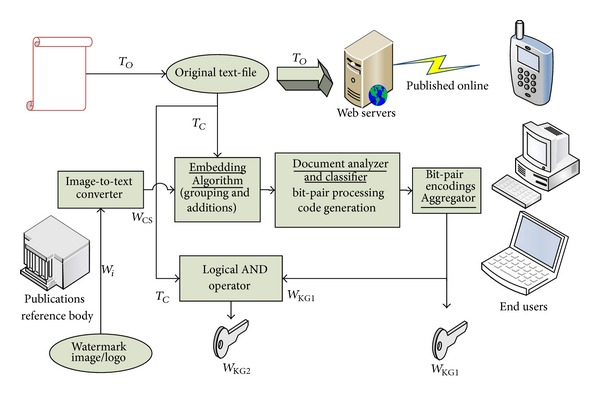
Watermark encoding process.

The embedding process (Figure 4) begins with an image logo, *W*_*I*_, being converted into a character sequence, *W*_CS_, and embedded in a copy of the cover document, *T*_*C*_. The* image-to-text converter* block at the encoder can be generalised/replaced with other media converters and therefore made applicable to any multimedia input or digital information that converts the data into a binary string prior to the encoding process. Meanwhile, the original document, *T*_*O*_, is unaltered and sent for online dissemination. The watermark logo, *W*_*I*_, is the unique signature of an organization/publisher or individual that owns rights to the digital content/online document. The embedding phase is based on a spread-spectrum technique that inserts one-watermark character per set (insertions only into the first word of each set), with the set size, *S*, being set to two words, forming a word pair. The result of the embedding is then passed for processing within the document analyzer and classifier (DAC), which uses the Unicode standard to numerate the words into binary Unicode summations (sum_*j*_for the first word and sum_*j*+1  _ for the second word) for further processing. Next, we use a logical XOR operation/function of the *k*th bit-positions of both words in each word pair set to produce an (*F*_*k*_) function code for each of the bit positions.

**Figure 5:**
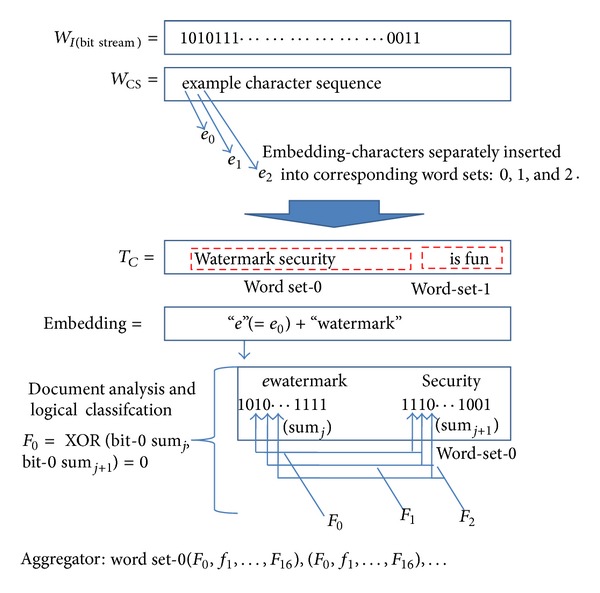
Partial function code generated in the encoder.

An example of generating a partial function code from *W*_*I*_ and *T*_*C*_ is illustrated in Figure 5 (example bit sequences shown may not be representative of actual words used).

The example in Figure 5 shows the publishers logo, *W*_*I*_, in binary format before being converted into the corresponding character sequence, *W*_CS_. Each of the embedding characters (*e*_*i*_) is then embedded into the first words of each word set in *T*_*C*_ with *e*_0_ being embedded into the first word of the first word set and *e*_1_ being embedded into the first word of the second word set and so on.

One of the main components in the encoding process is the use of the DAC, which is comprised of a document-analysis phase and a bit-pair classifier. The DAC consists of two main components: the* analyzer* which converts each word into Unicode summations and a logical-XOR* classifier* of similar bit positions of adjacent words. The* document analyzer* is used for the conversion of each word in the cover text into a binary summation of its constituent characters, whereas the* classifier* passes through the document, sampling similar bit positions in adjacent words of each set and producing a one-bit result of the XOR operation, an (*F*_*k*_) function code for each bit position operation between the two words. Similar function codes are then generated for the remaining bit-positions in the set. It is assumed that after all necessary summation operations, each word is represented using a 17-bit binary result. In this algorithm, two 16-bit Unicode values, as in the standard Unicode table [32], were added together, which produces a 17-bit result in the case of an overflow. Hence, each word set allocated 17-bit storage/memory to contain the result of the addition operation from the previous step. The DAC operation in the example of Figure 5 is shown to produce an *F*-result bit sequence through the logical operations on each bit position of the words in each set, before being output and aggregated.

The DAC processes each set in turn, with each word pair in a set being clearly separated by spaces, and words are assumed to begin with only nonspace characters. During the analysis phase, spaces are considered as part of the previous word encountered. By passing through the entire document, the* classifier* would be responsible for generating the individual function codes which are then used as input to the aggregator to produce a unique key* W*_KG1_. In the algorithm proposed in this paper, all logical and comparison operations are performed on Unicode binary values to extend our approach to all Unicode-supported languages. Next, a logical AND operation using the* W*_KG1_ and *T*_*C*_ is used to generate a second unique key,* W*_KG2_, as shown in Figure 4. Notably, all input characters (from *T*_*C*_ and *W*_CS_) were padded to 16-bit Unicode values, ensuring that logical operations (like ADD during the DAC stage or in the last/AND stage between* W*_KG1_ and *T*_*C*_) result with no loss in the 17-bit results generated. Finally, the two keys generated, together with the original document and time-stamped logo, are registered with a certification authority (CA)—a trusted third-party intermediary body in the digital community.

**Figure 6:**
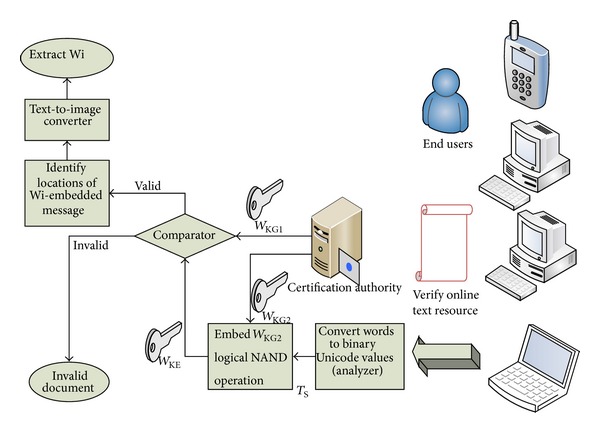
Watermark decoding process.

Enquiries pertaining to document authenticity, source tracing, and tamper detection tests of an online document are addressed using the decoding process, whereby the document under scrutiny, *T*_*S*_, is passed for processing, in which the* analyzer* algorithm converts each word into binary Unicode values. This proceeds with CA embedding the stored signature/key,* W*_KG2_, into the output produced by the* analyzer* using a NAND operation, the result of which is passed to the* comparator*. Simultaneously, CA passes the unique key,* W*_KG1_, into the comparator for equivalence testing (*w*) between the* W*_KG1_ and *W*_KE_. If the document is valid, the decoder extracts the characters (*e*_0_⋯*e*_*i*_) of the embedded *W*_CS_ character stream and converts the embedded data into a watermark image, *W*_*I*_, (using a text-to-image convertor) which thereby identifies the true owner. The details of the proposed decoder are shown in Figure 6.

In the proposed system, the document owner/publisher is responsible for generating the watermark keys/signatures (*W*_KG1_ and* W*_KG2_) and registering the time-stamped key, logo, and algorithm with the CA, whilst the CA is responsible for decoding the digital content and examining the watermark during the verification/decoding process for purposes of authenticity and source verification upon client requests. Hence, the correct keys (to be stored at the CA) required for verification checking at the client side can only be generated from the known publisher given that the original document is used as input to the encoder. Furthermore, the algorithm is only required by the publisher (and not the CA or client side) and hence is not made public.

### 3.2. Encoder and Decoder Algorithm Design

The watermark encoding and decoding algorithms are presented in Algorithms 1 and 2.

### 3.3. Design Issues and Advantages

The approach proposed in this paper ensures that the hybrid logical-watermark concept remains intact and valid in the following scenarios:

- when the font style, size, colour, and so forth are modified;
- when the whole document is copied (e.g., transported) onto another empty or nonempty document;
- when document integrity remains robust in the face of OCR techniques and exact retyping with the support of the standard Unicode format;
- when the detected watermark cannot be destroyed without distortion and therefore invalidating the document at the end user.

Furthermore, the logical watermark is characterized by the following.

- It cannot be detected, derived, or extracted from the host document, therefore achieving 100% imperceptibility.
- There is no additional increase to the original file size.
- A partial copy of the document does not allow the watermark to be detected.
- Scrutinizing the authenticity of a document in question can be performed by extracting/detecting the watermark to prove the rightful author.
- During the detection process, tampered documents may be evaluated as traceable to an original source based on the “*closeness measure,*” which measures the degree of similarity (e.g., as in the ratio of similar bits) of the extracted/recovered watermark image with the closest CA-registered watermark image. This in turn may be used to identify the locations of the modified bits in the document.
- Our encoding method supports circularly embedding of the watermark image in the document allowing for increased robustness and tamper detection abilities, since the watermark can be extracted from multiple segments of the document and compared for locating modified characters.

A drawback of this approach is evident in the space required at the CA's database for storing the keys W_KG1_ and W_KG2_, generated at the encoder side. In this study, a set size of 2 was considered (as an inner parameter), which for a document of 20,000 words requires 10,000 word sets ∗ 17 bits per word set = 170,000 bits of storage at the CA. This problem of large storage requirements can be addressed since the encoder design enables the set size to be readjusted at the publisher side (since it is only a fixed-value input to the encoder algorithm) to accommodate the CA's space limitations when necessary.

## 4. Results Analysis/Summary of Results

This section provides results and analysis of the proposed logical-watermarking approach in terms of our computational cost and application-driven cost-function requirements:* imperceptibility* and* document-integrity robustness* for authenticity and tamper detection. The* imperceptibility* requirement is addressed given that no one other than the owner and CA can know about the existence of any watermark in the document since the original text, *T*_*O*_, is unchanged after encoding. Consequently, unauthorized parties are not able to detect any existing watermarks, thereby reducing the probability of attacks or tampering via the communications channel. The* document-integrity robustness* requirement is essential for document authenticity and tamper detection and is addressed by detecting any change in the original document (e.g., at the comparator stage) as when the document has been subject to third-party modifications which would invalidate the document for end users. Notably, our design had enabled the retrieval of the original publisher logo, following the validity decision in the decoder using a function code generation scheme that allows us to obtain the embedded *W*_*I*_-bits at the encoding phase. Furthermore, as explained in the “*design issues*” discussion (Section 3.3) above, our encoder supports accumulative (circular) watermark embedding which had also resulted in increased robustness.

On the other hand, traditional text-watermarking involves embedding a watermark through the modification of document layout and appearance therefore possessing poor robustness since they cannot recover the watermark following simple formatting operations on the document [14]. The numerical results pertaining to real-sample tests are highlighted in Table 1, with the analysis and discussion of the benefits and features of those approaches being presented in Table 2. In Tables 1 and 2, the proposed method is compared with traditional format-encoding based watermarking and text-based zero-watermarking methods from the literature.

Our encoding and decoding algorithms were implemented in C++. The programs were compiled by a C++ compiler of version GCC 4.8 under the Linux operating system* Ubuntu 11.10*. Tests were run on a Pentium i3 processor of 1.7 GHz. The computational times were computed using the standard C function* clock()* which requires the header file* ctime*. The following C++ code fragment was used to calculate the execution time of an algorithm in seconds:

 const clock_t beginTime = clock();
 //encoding/decoding algorithm;
 double computationalTime = double (clock () − beginTime)/CLOCKS_PER_SEC.

In Table 1, five sample text* files* were used in all computations of encoder and decoder times for the algorithms being compared; those had consisted of algorithms [33–36] and our new proposed algorithm in this paper. Each of the encoder and decoder computational times was then calculated for each of the sample text files arranged in increasing order of size. Additionally, an average computational time per character metric (ave./character) was calculated for each algorithm to provide an indication of the average delay performances over various input text sizes. Table 1 shows that our proposed approach is very comparable with the existing approaches in terms of computational cost requirements. At the encoder stage, our proposed method had provided improvements over the works in [34, 35] and Method B from [36], whilst performing approximately equal to the approach in [33] and less than that of Method A [36]. Meanwhile at the decoder stage, the proposed method had produced results better than that of [34, 35] and longer times than those of [33, 36] due to the additional times required in identifying tampered locations and/or extracting the publisher's logo when needed.

Next, a comparison of the features and benefits between traditional watermarking methods, prior zero-watermarking methods [1, 33–36], and the new proposed watermarking method is now examined in detail in Table 2. A number of frequently recurring performance metrics and concepts from the digital-watermarking literature were considered as the basis for our comparisons. Those comparative metrics had included properties such as overhead complexity, embedding type, effect of watermark embedding on the original file, compatibility, and language dependencies. Moreover, a comparison of some powerful capabilities had included authenticity verification, tamper detection/identification, and the degree of robustness, imperceptibility, and capacity. Finally, each algorithm's ability to extract the owner's/publisher's watermark logo at the client side is presented in the final row of Table 2.

From Table 2, numerous improvements are identified by comparing the proposed approach in this paper (e.g., the rightmost column) with that of other traditional approaches from the literature. Specifically, it is shown that key advantages are gained over traditional approaches in terms of overhead, watermark transportability, compatibility, document authenticity verification, and tamper detection capabilities. Hence, the proposed approach had resulted in improvements in the key performance metrics of integrity robustness, imperceptibility, and capacity ratio (last three rows in Table 2). Furthermore, several outstanding improvements were also evident when comparing our proposed approach with existing zero-watermarking approaches from the literature. For instance, key enhancements were observed in format-compatibility, language independency, tamper detection, identification capabilities, and finally, through its ability to extract the publishers watermark logo. From Table 2, further benefits are also evident over traditional approaches, such as imperceptibility performance and integrity robustness.

From Table 2, the advantages of our approach are also highlighted in the final approach (shown in the rightmost column). In all approaches considered, the overhead parameter had only referred to the overhead at the publisher/encoder side. In Section 3, a discussion was given on the overhead of our approach on the CA. Notably, the work in [33, 36] was closely matched in most advantages except that our new approach here has the ability to exactly extract the publisher's watermark logo rather than simply determine whether the text-under-scrutiny (*T*_*S*_—from the decoder-side) is valid or not. Significantly, our new approach was able to localise tamper attempts performed on *T*_*S*_, an improvement not previously found in the related work [33, 35, 36]. These contrasting features that improve the work from [33] are observed in the last row and the fifth last row in Table 2.

The proposed technique is a new zero-watermarking approach which can deal with sensitive documents. From Tables 1 and 2, it is noted that the proposed method may not outperform other methods in terms of computational time for the encoding and decoding phases; however, compared to other methods the proposed method addresses some of the weaknesses found in the current available techniques; that is, it is not language-dependent; it has tamper detection and identification capabilities; it is robust and capable of extracting publishers watermark logo.

## 5. Discussion and Conclusion

The proliferation and expansion of the Internet suggest that more attention is required for the security and protection of online multimedia data and particularly for the dominant text medium. Many existing text-watermarking algorithms lack the requirements of robustness, imperceptibility, and document authenticity verification. This paper has proposed a novel hybrid approach involving concepts from digital signatures and logical text watermarking independent of the underlying language, given that it can be encoded in standard Unicode. The proposed algorithm can be used to protect electronic documents and digital textual content from tampering, forgery, and illegal content manipulation, whilst removing many implementation redundancies and complexities found in previous schemes. Additionally, the proposed approach can achieve effective protection and authenticity verification, while its computational costs and quality of results obtained are completely practical. The drawbacks that are being considered for improvement in future work involve reducing storage requirements at the CA and further enhancing computational times, both of which become more significant for very large text document samples. Significant contributions of this paper include introduction of a new design framework for a text-based logical watermarking scheme, a mechanism for adapting and optimizing the framework for specific target-applications, and finally demonstrating how such an approach can bypass the menace of most publishers' watermark targeted attacks by avoiding all such physical and vulnerable/suspicious modifications in the text due to the encoding process.

Future work and open research issues have emerged as a result of this work and primarily involve, first, testing our approach with larger varieties of sensitive online document samples and enhancing the proposed approach to become a commercially viable solution, to be developed as an essential tool for reference/certification bodies/organizations concerned with the dissemination of sensitive/critical text resources. Second, the planned next phase of this work considers evaluating language-specific embedding characters and their benefits on our performance metrics of interest and whether they can be used to enhance our cost parameters. Other opportunities for future work involve adapting our approach to the other major applications of text-watermarking, namely, copyright protection of text documents, by comparing the recovered/decoded watermark from the illegally copied document with the watermarks stored at the CA, in terms of their* degrees-of-similarity*. Finally, it is also anticipated that this work will open new research directions aimed at developing and advancing the state of the art in multimedia-based logical watermarking in the two major application domains of copyright protection and authenticity verification/tamper detection.

## Figures and Tables

**Algorithm 1:**
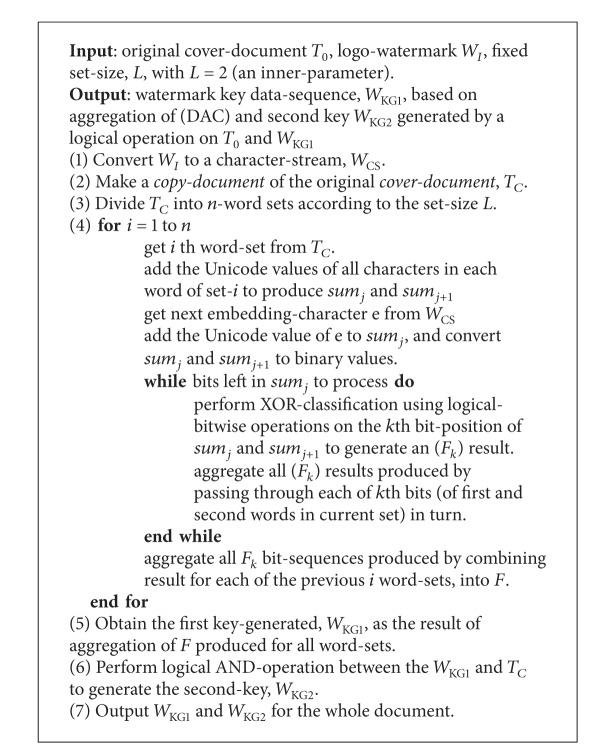
Encoding algorithm.

**Algorithm 2:**
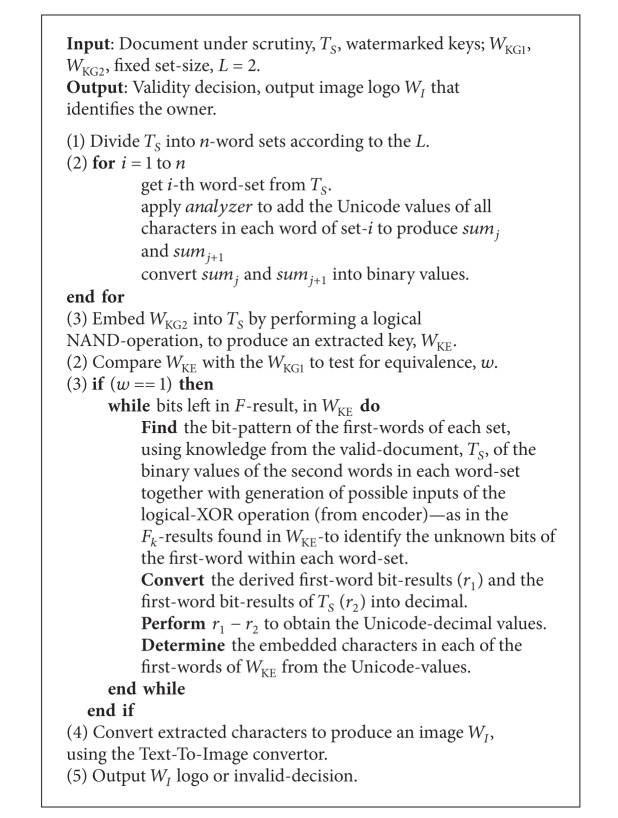
Decoding algorithm.

**Table 1:** Computational-cost comparison between relevant approaches.

File name	No. of Chars	Computational time [encoder (ms)]						Computational time [decoder (ms)]					
		(Tayan et al., [33])	(Jalil et al., [34])	(Meng et al., [35])	Method A from [36]	Method B from [36]	Proposed approach	(Tayan et al., [33])	(Jalil et al., [34])	(Meng et al., [35])	Method A from [36]	Method B from [36]	Proposed approach
Text 1	28915	30	180	210	20	40	**20**	20	170	224	10	20	**20**
Text 2	47974	40	160	350	30	60	**40**	30	240	371	20	40	**40**
Text 3	54839	60	195	410	50	100	**60**	40	278	460	40	40	**70**
Text 4	116794	80	1714	1850	70	190	**100**	70	1684	1891	60	80	**110**
Text 5	166166	130	590	620	120	300	**160**	100	520	640	100	130	**170**
Ave./char		0.00089	0.0063	0.0083	0.00071	0.0016	**0.00089**	0.00065	0.0067	0.0088	0.00052	0.00074	**0.00095**

**Table 2:** Comparison of features and benefits between watermarking methods.

Concept/metric	Traditional watermark approaches	Zero-watermarking approach [1]	Zero-watermarking approach [35]	Zero-watermarking approach [34]	Zero-watermarking approach [33, 36]	Proposed watermarking method
Overhead (additional)	Proportional to embedded key size	None	None	None	None	None

Embedding mode	Format-encoding	Logical: zero-watermark encoding	Logical: zero-watermark encoding	Logical: zero-watermark encoding	Logical: zero-watermark encoding	Logical: zero-watermark encoding

Location of watermark message (*W*_*M*_)	Embedded in *T*_*O*_	Embedded in *T*_*C*_	Embedded in *T*_*C*_	Embedded in *T*_*C*_	Embedded in *T*_*C*_	Embedded in *T*_*C*_

Processing and embedding decision	Based on searching through text for candidate words, lines, and spaces	Based on double-letter words in English language	Based on sentence entropy	Based on the first letter with specific word lengths	Based on comparing Unicode summations	Based on comparing Unicode summations and logical operations

Compatible with various formats?	Limited	Yes, only English character support needed	Supports Chinese language only	Supports English language only	Yes, host-document language character support/Unicode needed	**Yes, host-document language character support/Unicode needed**

Language-dependent	No	Yes	Yes	Yes	No	**No**

Document- authenticity verification	No	Yes	Yes	Yes	Yes	Yes

Tamper-detection and identification capabilities	No	No	No	Yes	No	**Yes**

Integrity robustness	Weak	Strong	Strong	Strong	Strong	Strong

Perceptibility performance	Low-medium	High	High	High	High	High

Capacity ratio performance	Inversely proportional to perceptual similarity	High	High	High	High	High

Capability to extract publishers watermark logo	No	Yes	No	No	No	**Yes**
